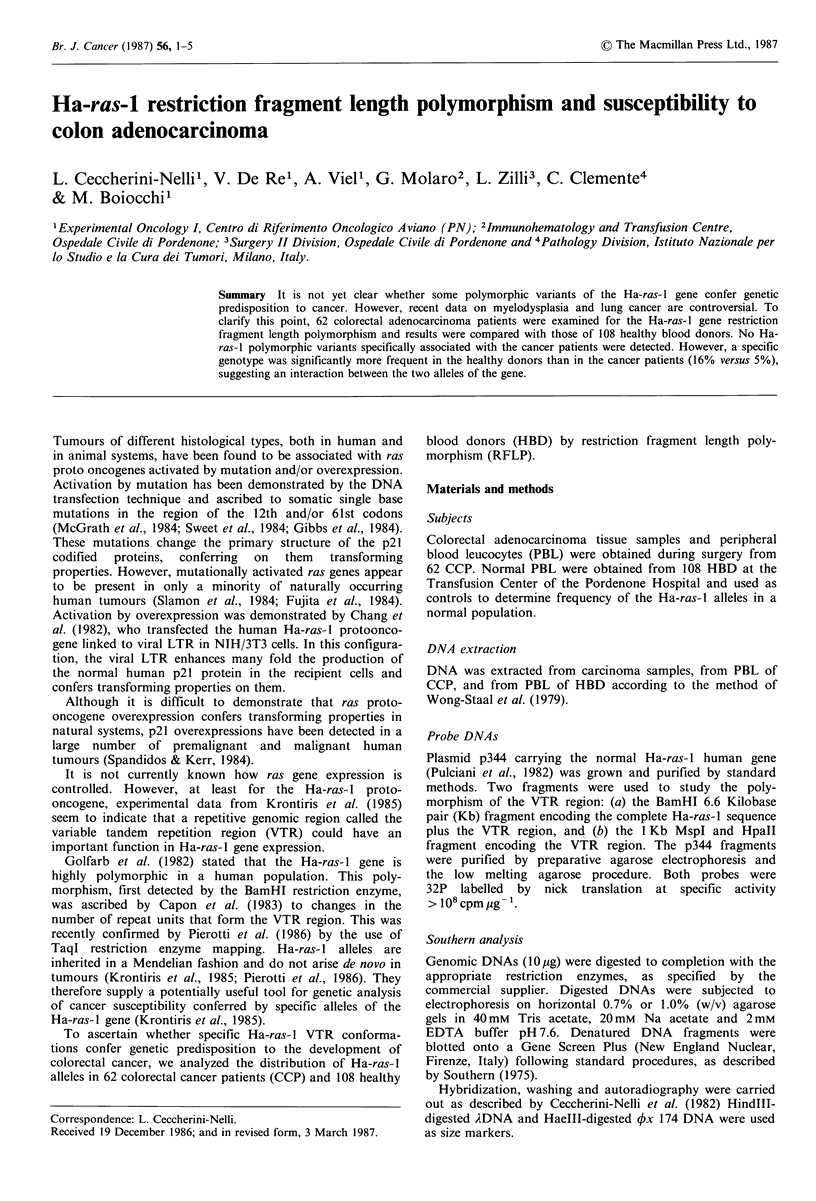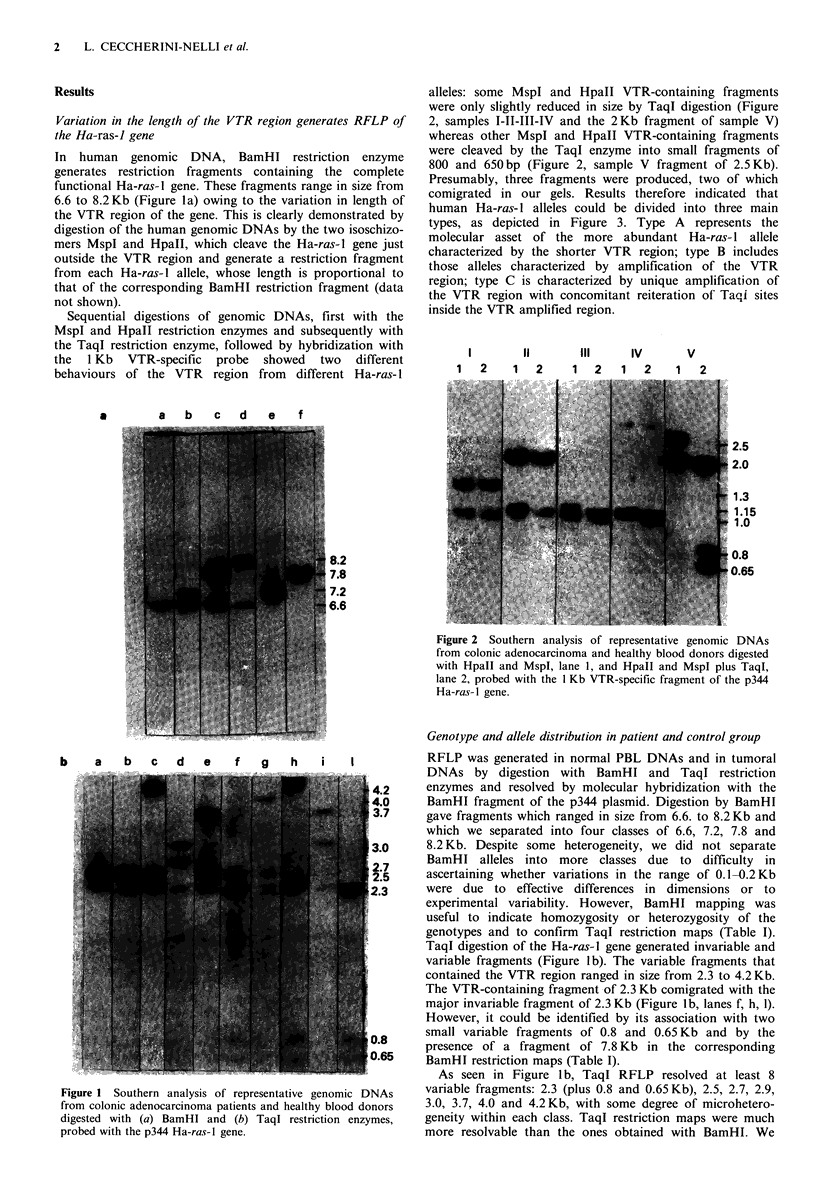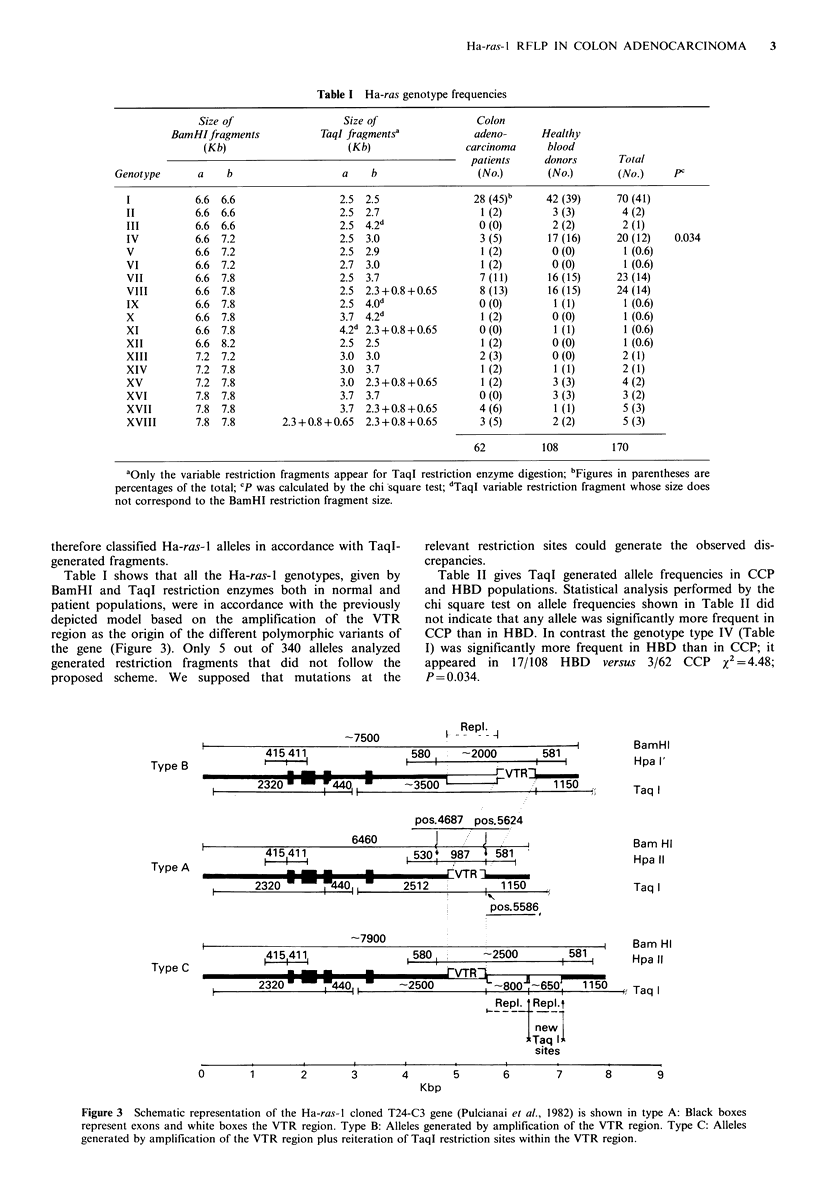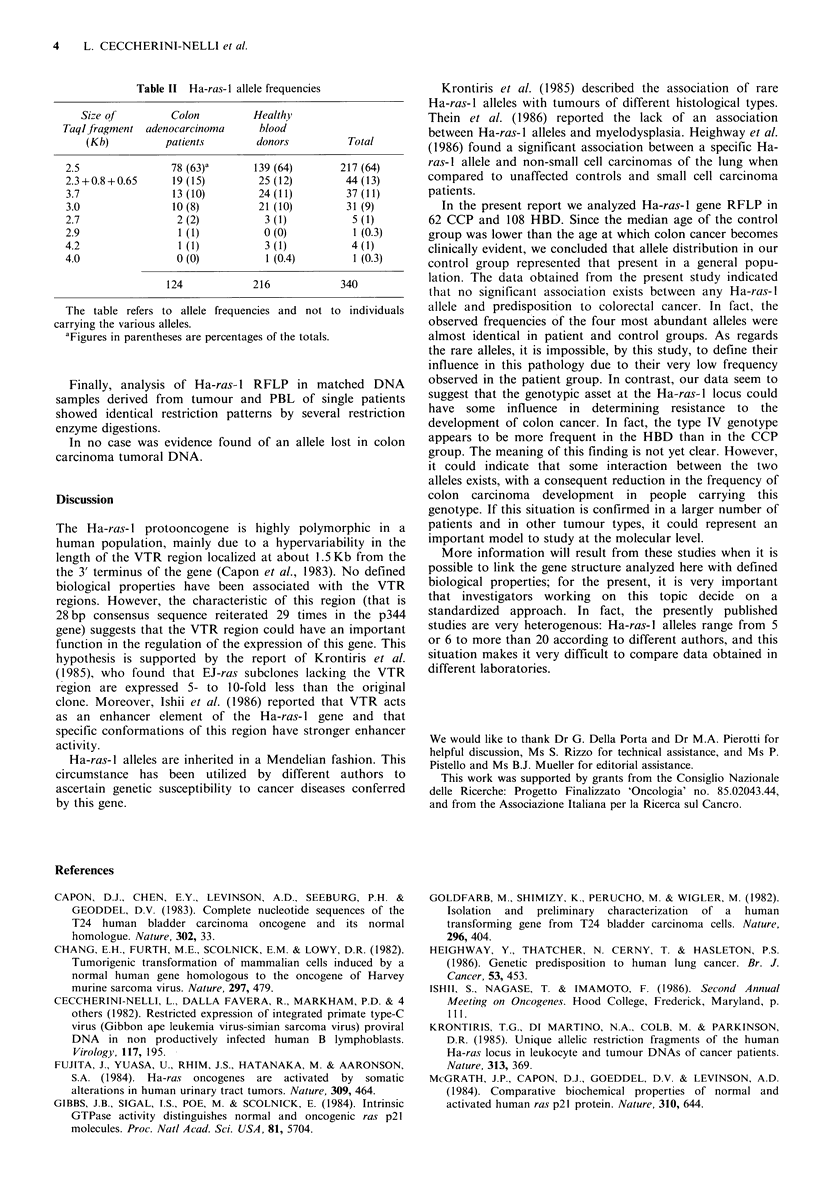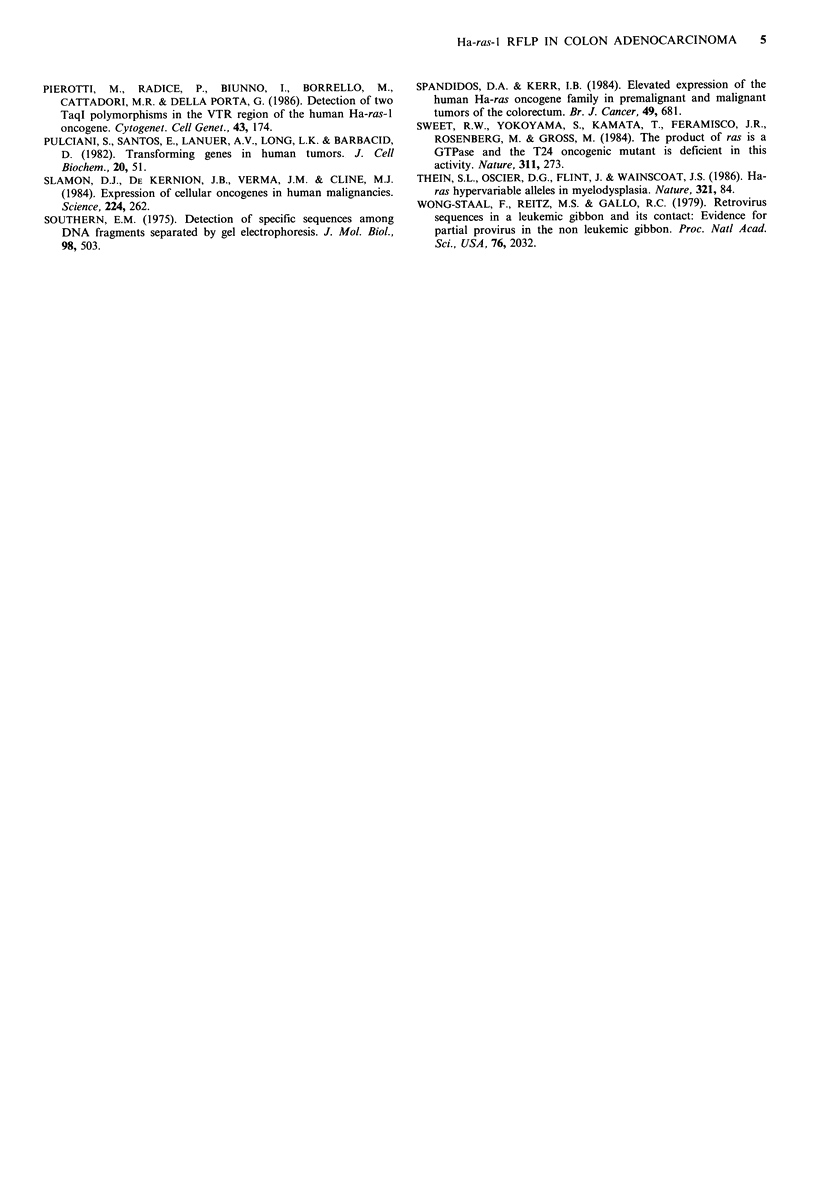# Ha-ras-1 restriction fragment length polymorphism and susceptibility to colon adenocarcinoma.

**DOI:** 10.1038/bjc.1987.143

**Published:** 1987-07

**Authors:** L. Ceccherini-Nelli, V. De Re, A. Viel, G. Molaro, L. Zilli, C. Clemente, M. Boiocchi

## Abstract

**Images:**


*Br. J. Cancer* (1987) **56**, 1–5 © The Macmillan Press Ltd., 1987

### Ha-*ras*-1 restriction fragment length polymorphism and susceptibility to colon adenocarcinoma

L. Ceccherini-Nelli[1], V. De Re[1], A. Viel[1], G. Molaro[2], L. Zilli[3], C. Clemente[4] & M. Boiocchi[1]

[1]*Experimental Oncology I, Centro di Riferimento Oncologico Aviano (PN);* [2]*Immunohematology and Transfusion Centre, Ospedale Civile di Pordenone;* [3]*Surgery II Division, Ospedale Civile di Pordenone and* [4]*Pathology Division, Istituto Nazionale per lo Studio e la Cura dei Tumori, Milano, Italy.*

**Summary** It is not yet clear whether some polymorphic variants of the Ha-*ras*-1 gene confer genetic predisposition to cancer. However, recent data on myelodysplasia and lung cancer are controversial. To clarify this point, 62 colorectal adenocarcinoma patients were examined for the Ha-*ras*-1 gene restriction fragment length polymorphism and results were compared with those of 108 healthy blood donors. No Ha-*ras*-1 polymorphic variants specifically associated with the cancer patients were detected. However, a specific genotype was significantly more frequent in the healthy donors than in the cancer patients (16% *versus* 5%), suggesting an interaction between the two alleles of the gene.

Tumours of different histological types, both in human and in animal systems, have been found to be associated with *ras* proto oncogenes activated by mutation and/or overexpression. Activation by mutation has been demonstrated by the DNA transfection technique and ascribed to somatic single base mutations in the region of the 12th and/or 61st codons (McGrath *et al.*, 1984; Sweet *et al.*, 1984; Gibbs *et al.*, 1984). These mutations change the primary structure of the p21 codified proteins, conferring on them transforming properties. However, mutationally activated *ras* genes appear to be present in only a minority of naturally occurring human tumours (Slamon *et al.*, 1984; Fujita *et al.*, 1984). Activation by overexpression was demonstrated by Chang *et al.* (1982), who transfected the human Ha-*ras*-1 protooncogene linked to viral LTR in NIH/3T3 cells. In this configuration, the viral LTR enhances many fold the production of the normal human p21 protein in the recipient cells and confers transforming properties on them.

Although it is difficult to demonstrate that *ras* proto-oncogene overexpression confers transforming properties in natural systems, p21 overexpressions have been detected in a large number of premalignant and malignant human tumours (Spandidos & Kerr, 1984).

It is not currently known how *ras* gene expression is controlled. However, at least for the Ha-*ras*-1 proto-oncogene, experimental data from Krontiris *et al.* (1985) seem to indicate that a repetitive genomic region called the variable tandem repetition region (VTR) could have an important function in Ha-*ras*-1 gene expression.

Golfarb *et al.* (1982) stated that the Ha-*ras*-1 gene is highly polymorphic in a human population. This polymorphism, first detected by the BamHI restriction enzyme, was ascribed by Capon *et al.* (1983) to changes in the number of repeat units that form the VTR region. This was recently confirmed by Pierotti *et al.* (1986) by the use of TaqI restriction enzyme mapping. Ha-*ras*-1 alleles are inherited in a Mendelian fashion and do not arise *de novo* in tumours (Krontiris *et al.*, 1985; Pierotti *et al.*, 1986). They therefore supply a potentially useful tool for genetic analysis of cancer susceptibility conferred by specific alleles of the Ha-*ras*-1 gene (Krontiris *et al.*, 1985).

To ascertain whether specific Ha-*ras*-1 VTR conformations confer genetic predisposition to the development of colorectal cancer, we analyzed the distribution of Ha-*ras*-1 alleles in 62 colorectal cancer patients (CCP) and 108 healthy blood donors (HBD) by restriction fragment length polymorphism (RFLP).

### Materials and methods

### *Subjects*

Colorectal adenocarcinoma tissue samples and peripheral blood leucocytes (PBL) were obtained during surgery from 62 CCP. Normal PBL were obtained from 108 HBD at the Transfusion Center of the Pordenone Hospital and used as controls to determine frequency of the Ha-*ras*-1 alleles in a normal population.

### *DNA extraction*

DNA was extracted from carcinoma samples, from PBL of CCP, and from PBL of HBD according to the method of Wong-Staal *et al.* (1979).

### *Probe DNAs*

Plasmid p344 carrying the normal Ha-*ras*-1 human gene (Pulciani *et al.*, 1982) was grown and purified by standard methods. Two fragments were used to study the polymorphism of the VTR region: (*a*) the BamHI 6.6 Kilobase pair (Kb) fragment encoding the complete Ha-*ras*-1 sequence plus the VTR region, and (*b*) the 1 Kb MspI and HpaII fragment encoding the VTR region. The p344 fragments were purified by preparative agarose electrophoresis and the low melting agarose procedure. Both probes were 32P labelled by nick translation at specific activity $>10^8$ cpm $\mu g^{-1}$.

### *Southern analysis*

Genomic DNAs (10 $\mu$g) were digested to completion with the appropriate restriction enzymes, as specified by the commercial supplier. Digested DNAs were subjected to electrophoresis on horizontal 0.7% or 1.0% (w/v) agarose gels in 40 mM Tris acetate, 20 mM Na acetate and 2 mM EDTA buffer pH 7.6. Denatured DNA fragments were blotted onto a Gene Screen Plus (New England Nuclear, Firenze, Italy) following standard procedures, as described by Southern (1975).

Hybridization, washing and autoradiography were carried out as described by Ceccherini-Nelli *et al.* (1982) HindIII-digested $\lambda$DNA and HaeIII-digested $\phi x$ 174 DNA were used as size markers.

Correspondence: L. Ceccherini-Nelli.
Received 19 December 1986; and in revised form, 3 March 1987.

### Results

### *Variation in the length of the VTR region generates RFLP of the Ha-*ras*-1 gene*

In human genomic DNA, BamHI restriction enzyme generates restriction fragments containing the complete functional Ha-*ras*-1 gene. These fragments range in size from 6.6 to 8.2 Kb (Figure 1a) owing to the variation in length of the VTR region of the gene. This is clearly demonstrated by digestion of the human genomic DNAs by the two isoschizomers MspI and HpaII, which cleave the Ha-*ras*-1 gene just outside the VTR region and generate a restriction fragment from each Ha-*ras*-1 allele, whose length is proportional to that of the corresponding BamHI restriction fragment (data not shown).

Sequential digestions of genomic DNAs, first with the MspI and HpaII restriction enzymes and subsequently with the TaqI restriction enzyme, followed by hybridization with the 1 Kb VTR-specific probe showed two different behaviours of the VTR region from different Ha-*ras*-1 alleles: some MspI and HpaII VTR-containing fragments were only slightly reduced in size by TaqI digestion (Figure 2, samples I-II-III-IV and the 2 Kb fragment of sample V) whereas other MspI and HpaII VTR-containing fragments were cleaved by the TaqI enzyme into small fragments of 800 and 650 bp (Figure 2, sample V fragment of 2.5 Kb). Presumably, three fragments were produced, two of which comigrated in our gels. Results therefore indicated that human Ha-*ras*-1 alleles could be divided into three main types, as depicted in Figure 3. Type A represents the molecular asset of the more abundant Ha-*ras*-1 allele characterized by the shorter VTR region; type B includes those alleles characterized by amplification of the VTR region; type C is characterized by unique amplification of the VTR region with concomitant reiteration of TaqI sites inside the VTR amplified region.

**Figure 1** Southern analysis of representative genomic DNAs from colonic adenocarcinoma patients and healthy blood donors digested with (*a*) BamHI and (*b*) TaqI restriction enzymes, probed with the p344 Ha-*ras*-1 gene.

**Figure 2** Southern analysis of representative genomic DNAs from colonic adenocarcinoma and healthy blood donors digested with HpaII and MspI, lane 1, and HpaII and MspI plus TaqI, lane 2, probed with the 1 Kb VTR-specific fragment of the p344 Ha-*ras*-1 gene.

### *Genotype and allele distribution in patient and control group*

RFLP was generated in normal PBL DNAs and in tumoral DNAs by digestion with BamHI and TaqI restriction enzymes and resolved by molecular hybridization with the BamHI fragment of the p344 plasmid. Digestion by BamHI gave fragments which ranged in size from 6.6. to 8.2 Kb and which we separated into four classes of 6.6, 7.2, 7.8 and 8.2 Kb. Despite some heterogeneity, we did not separate BamHI alleles into more classes due to difficulty in ascertaining whether variations in the range of 0.1–0.2 Kb were due to effective differences in dimensions or to experimental variability. However, BamHI mapping was useful to indicate homozygosity or heterozygosity of the genotypes and to confirm TaqI restriction maps (Table I). TaqI digestion of the Ha-*ras*-1 gene generated invariable and variable fragments (Figure 1b). The variable fragments that contained the VTR region ranged in size from 2.3 to 4.2 Kb. The VTR-containing fragment of 2.3 Kb comigrated with the major invariable fragment of 2.3 Kb (Figure 1b, lanes f, h, l). However, it could be identified by its association with two small variable fragments of 0.8 and 0.65 Kb and by the presence of a fragment of 7.8 Kb in the corresponding BamHI restriction maps (Table I).

As seen in Figure 1b, TaqI RFLP resolved at least 8 variable fragments: 2.3 (plus 0.8 and 0.65 Kb), 2.5, 2.7, 2.9, 3.0, 3.7, 4.0 and 4.2 Kb, with some degree of microheterogeneity within each class. TaqI restriction maps were much more resolvable than the ones obtained with BamHI. We

**Table I** Ha-*ras* genotype frequencies

| Genotype | Size of BamHI fragments (Kb) a | b | Size of TaqI fragments[a] (Kb) a | b | Colon adeno-carcinoma patients (No.) | Healthy blood donors (No.) | Total (No.) | P[c] |
|---|---|---|---|---|---|---|---|---|
| I | 6.6 | 6.6 | 2.5 | 2.5 | 28 (45)[b] | 42 (39) | 70 (41) | |
| II | 6.6 | 6.6 | 2.5 | 2.7 | 1 (2) | 3 (3) | 4 (2) | |
| III | 6.6 | 6.6 | 2.5 | 4.2[d] | 0 (0) | 2 (2) | 2 (1) | |
| IV | 6.6 | 7.2 | 2.5 | 3.0 | 3 (5) | 17 (16) | 20 (12) | 0.034 |
| V | 6.6 | 7.2 | 2.5 | 2.9 | 1 (2) | 0 (0) | 1 (0.6) | |
| VI | 6.6 | 7.2 | 2.7 | 3.0 | 1 (2) | 0 (0) | 1 (0.6) | |
| VII | 6.6 | 7.8 | 2.5 | 3.7 | 7 (11) | 16 (15) | 23 (14) | |
| VIII | 6.6 | 7.8 | 2.5 | 2.3+0.8+0.65 | 8 (13) | 16 (15) | 24 (14) | |
| IX | 6.6 | 7.8 | 2.5 | 4.0[d] | 0 (0) | 1 (1) | 1 (0.6) | |
| X | 6.6 | 7.8 | 3.7 | 4.2[d] | 1 (2) | 0 (0) | 1 (0.6) | |
| XI | 6.6 | 7.8 | 4.2[d] | 2.3+0.8+0.65 | 0 (0) | 1 (1) | 1 (0.6) | |
| XII | 6.6 | 8.2 | 2.5 | 2.5 | 1 (2) | 0 (0) | 1 (0.6) | |
| XIII | 7.2 | 7.2 | 3.0 | 3.0 | 2 (3) | 0 (0) | 2 (1) | |
| XIV | 7.2 | 7.8 | 3.0 | 3.7 | 1 (2) | 1 (1) | 2 (1) | |
| XV | 7.2 | 7.8 | 3.0 | 2.3+0.8+0.65 | 1 (2) | 3 (3) | 4 (2) | |
| XVI | 7.8 | 7.8 | 3.7 | 3.7 | 0 (0) | 3 (3) | 3 (2) | |
| XVII | 7.8 | 7.8 | 3.7 | 2.3+0.8+0.65 | 4 (6) | 1 (1) | 5 (3) | |
| XVIII | 7.8 | 7.8 | 2.3+0.8+0.65 | 2.3+0.8+0.65 | 3 (5) | 2 (2) | 5 (3) | |
| | | | | | 62 | 108 | 170 | |

[a]Only the variable restriction fragments appear for TaqI restriction enzyme digestion; [b]Figures in parentheses are percentages of the total; [c]*P* was calculated by the chi square test; [d]TaqI variable restriction fragment whose size does not correspond to the BamHI restriction fragment size.

therefore classified Ha-*ras*-1 alleles in accordance with TaqI-generated fragments.

Table I shows that all the Ha-*ras*-1 genotypes, given by BamHI and TaqI restriction enzymes both in normal and patient populations, were in accordance with the previously depicted model based on the amplification of the VTR region as the origin of the different polymorphic variants of the gene (Figure 3). Only 5 out of 340 alleles analyzed generated restriction fragments that did not follow the proposed scheme. We supposed that mutations at the relevant restriction sites could generate the observed discrepancies.

Table II gives TaqI generated allele frequencies in CCP and HBD populations. Statistical analysis performed by the chi square test on allele frequencies shown in Table II did not indicate that any allele was significantly more frequent in CCP than in HBD. In contrast the genotype type IV (Table I) was significantly more frequent in HBD than in CCP; it appeared in 17/108 HBD *versus* 3/62 CCP $\chi^2=4.48$; $P=0.034$.

**Figure 3** Schematic representation of the Ha-*ras*-1 cloned T24-C3 gene (Pulcianai *et al*., 1982) is shown in type A: Black boxes represent exons and white boxes the VTR region. Type B: Alleles generated by amplification of the VTR region. Type C: Alleles generated by amplification of the VTR region plus reiteration of TaqI restriction sites within the VTR region.

**Table II** Ha-*ras*-1 allele frequencies

| *Size of TaqI fragment (Kb)* | *Colon adenocarcinoma patients* | *Healthy blood donors* | *Total* |
|---|---|---|---|
| 2.5 | 78 (63)[a] | 139 (64) | 217 (64) |
| 2.3+0.8+0.65 | 19 (15) | 25 (12) | 44 (13) |
| 3.7 | 13 (10) | 24 (11) | 37 (11) |
| 3.0 | 10 (8) | 21 (10) | 31 (9) |
| 2.7 | 2 (2) | 3 (1) | 5 (1) |
| 2.9 | 1 (1) | 0 (0) | 1 (0.3) |
| 4.2 | 1 (1) | 3 (1) | 4 (1) |
| 4.0 | 0 (0) | 1 (0.4) | 1 (0.3) |
| | 124 | 216 | 340 |

The table refers to allele frequencies and not to individuals carrying the various alleles.

[a]Figures in parentheses are percentages of the totals.

Finally, analysis of Ha-*ras*-1 RFLP in matched DNA samples derived from tumour and PBL of single patients showed identical restriction patterns by several restriction enzyme digestions.

In no case was evidence found of an allele lost in colon carcinoma tumoral DNA.

### Discussion

The Ha-*ras*-1 protooncogene is highly polymorphic in a human population, mainly due to a hypervariability in the length of the VTR region localized at about 1.5 Kb from the the 3′ terminus of the gene (Capon *et al.*, 1983). No defined biological properties have been associated with the VTR regions. However, the characteristic of this region (that is 28 bp consensus sequence reiterated 29 times in the p344 gene) suggests that the VTR region could have an important function in the regulation of the expression of this gene. This hypothesis is supported by the report of Krontiris *et al.* (1985), who found that EJ-*ras* subclones lacking the VTR region are expressed 5- to 10-fold less than the original clone. Moreover, Ishii *et al.* (1986) reported that VTR acts as an enhancer element of the Ha-*ras*-1 gene and that specific conformations of this region have stronger enhancer activity.

Ha-*ras*-1 alleles are inherited in a Mendelian fashion. This circumstance has been utilized by different authors to ascertain genetic susceptibility to cancer diseases conferred by this gene.

Krontiris *et al.* (1985) described the association of rare Ha-*ras*-1 alleles with tumours of different histological types. Thein *et al.* (1986) reported the lack of an association between Ha-*ras*-1 alleles and myelodysplasia. Heighway *et al.* (1986) found a significant association between a specific Ha-*ras*-1 allele and non-small cell carcinomas of the lung when compared to unaffected controls and small cell carcinoma patients.

In the present report we analyzed Ha-*ras*-1 gene RFLP in 62 CCP and 108 HBD. Since the median age of the control group was lower than the age at which colon cancer becomes clinically evident, we concluded that allele distribution in our control group represented that present in a general population. The data obtained from the present study indicated that no significant association exists between any Ha-*ras*-1 allele and predisposition to colorectal cancer. In fact, the observed frequencies of the four most abundant alleles were almost identical in patient and control groups. As regards the rare alleles, it is impossible, by this study, to define their influence in this pathology due to their very low frequency observed in the patient group. In contrast, our data seem to suggest that the genotypic asset at the Ha-*ras*-1 locus could have some influence in determining resistance to the development of colon cancer. In fact, the type IV genotype appears to be more frequent in the HBD than in the CCP group. The meaning of this finding is not yet clear. However, it could indicate that some interaction between the two alleles exists, with a consequent reduction in the frequency of colon carcinoma development in people carrying this genotype. If this situation is confirmed in a larger number of patients and in other tumour types, it could represent an important model to study at the molecular level.

More information will result from these studies when it is possible to link the gene structure analyzed here with defined biological properties; for the present, it is very important that investigators working on this topic decide on a standardized approach. In fact, the presently published studies are very heterogenous: Ha-*ras*-1 alleles range from 5 or 6 to more than 20 according to different authors, and this situation makes it very difficult to compare data obtained in different laboratories.

We would like to thank Dr G. Della Porta and Dr M.A. Pierotti for helpful discussion, Ms S. Rizzo for technical assistance, and Ms P. Pistello and Ms B.J. Mueller for editorial assistance.

This work was supported by grants from the Consiglio Nazionale delle Ricerche: Progetto Finalizzato 'Oncologia' no. 85.02043.44, and from the Associazione Italiana per la Ricerca sul Cancro.

### References

CAPON, D.J., CHEN, E.Y., LEVINSON, A.D., SEEBURG, P.H. & GEODDEL, D.V. (1983). Complete nucleotide sequences of the T24 human bladder carcinoma oncogene and its normal homologue. *Nature*, **302**, 33.

CHANG, E.H., FURTH, M.E., SCOLNICK, E.M. & LOWY, D.R. (1982). Tumorigenic transformation of mammalian cells induced by a normal human gene homologous to the oncogene of Harvey murine sarcoma virus. *Nature*, **297**, 479.

CECCHERINI-NELLI, L., DALLA FAVERA, R., MARKHAM, P.D. & 4 others (1982). Restricted expression of integrated primate type-C virus (Gibbon ape leukemia virus-simian sarcoma virus) proviral DNA in non productively infected human B lymphoblasts. *Virology*, **117**, 195.

FUJITA, J., YUASA, U., RHIM, J.S., HATANAKA, M. & AARONSON, S.A. (1984). Ha-*ras* oncogenes are activated by somatic alterations in human urinary tract tumors. *Nature*, **309**, 464.

GIBBS, J.B., SIGAL, I.S., POE, M. & SCOLNICK, E. (1984). Intrinsic GTPase activity distinguishes normal and oncogenic *ras* p21 molecules. *Proc. Natl Acad. Sci. USA*, **81**, 5704.

GOLDFARB, M., SHIMIZY, K., PERUCHO, M. & WIGLER, M. (1982). Isolation and preliminary characterization of a human transforming gene from T24 bladder carcinoma cells. *Nature*, **296**, 404.

HEIGHWAY, Y., THATCHER, N. CERNY, T. & HASLETON, P.S. (1986). Genetic predisposition to human lung cancer. *Br. J. Cancer*, **53**, 453.

ISHII, S., NAGASE, T. & IMAMOTO, F. (1986). *Second Annual Meeting on Oncogenes*. Hood College, Frederick, Maryland, p. 111.

KRONTIRIS, T.G., DI MARTINO, N.A., COLB, M. & PARKINSON, D.R. (1985). Unique allelic restriction fragments of the human Ha-*ras* locus in leukocyte and tumour DNAs of cancer patients. *Nature*, **313**, 369.

McGRATH, J.P., CAPON, D.J., GOEDDEL, D.V. & LEVINSON, A.D. (1984). Comparative biochemical properties of normal and activated human *ras* p21 protein. *Nature*, **310**, 644.

PIEROTTI, M., RADICE, P., BIUNNO, I., BORRELLO, M., CATTADORI, M.R. & DELLA PORTA, G. (1986). Detection of two TaqI polymorphisms in the VTR region of the human Ha-*ras*-1 oncogene. *Cytogenet. Cell Genet.*, **43**, 174.

PULCIANI, S., SANTOS, E., LANUER, A.V., LONG, L.K. & BARBACID, D. (1982). Transforming genes in human tumors. *J. Cell Biochem.*, **20**, 51.

SLAMON, D.J., DE KERNION, J.B., VERMA, J.M. & CLINE, M.J. (1984). Expression of cellular oncogenes in human malignancies. *Science*, **224**, 262.

SOUTHERN, E.M. (1975). Detection of specific sequences among DNA fragments separated by gel electrophoresis. *J. Mol. Biol.*, **98**, 503.

SPANDIDOS, D.A. & KERR, I.B. (1984). Elevated expression of the human Ha-*ras* oncogene family in premalignant and malignant tumors of the colorectum. *Br. J. Cancer*, **49**, 681.

SWEET, R.W., YOKOYAMA, S., KAMATA, T., FERAMISCO, J.R., ROSENBERG, M. & GROSS, M. (1984). The product of *ras* is a GTPase and the T24 oncogenic mutant is deficient in this activity. *Nature*, **311**, 273.

THEIN, S.L., OSCIER, D.G., FLINT, J. & WAINSCOAT, J.S. (1986). Ha-*ras* hypervariable alleles in myelodysplasia. *Nature*, **321**, 84.

WONG-STAAL, F., REITZ, M.S. & GALLO, R.C. (1979). Retrovirus sequences in a leukemic gibbon and its contact: Evidence for partial provirus in the non leukemic gibbon. *Proc. Natl Acad. Sci., USA*, **76**, 2032.